# Gender Distribution in Randomized Controlled Trials among Patients with Heart Failure with Reduced Ejection Fraction: A Systematic Review

**DOI:** 10.5334/gh.1093

**Published:** 2022-02-01

**Authors:** Asma Gulab, Hani Essa, Kevin Bryan Lo, Rajiv Sankaranarayanan, Janani Rangaswami

**Affiliations:** 1 Department of Medicine, Einstein Medical Center, PA, US; 2 Liverpool University Hospitals NHS Foundation Trust, Liverpool, UK; 3 Faculty of Life Sciences, University of Liverpool, GB; 4 Sidney Kimmel College of Thomas Jefferson University, PA, US

**Keywords:** Heart Failure, Gender disparaties, Randomized Controlled trials, Heatlh disparities, Heart Failure with Reduced EF

## Background

Heart failure (HF) is a major cause of morbidity and mortality at a global level, affecting 64 million people worldwide with about 40–50% of the hospitalized heart failure patients being female [1]. Despite high disease burden of HF in women, HF trials have consistently underrepresented women in enrollment [2]. The objective of this study was to determine the trends in the representation of women in randomized controlled trials (RCTs) for therapies for heart failure with reduced ejection fraction (HFrEF).

## Methods

This systematic review looked at the patterns and rates of inclusion of women in RCTs among patients with HFrEF. We utilized PubMed to extract RCTs conducted between January 1, 2010, and December 1, 2020. The medical subject headings (MeSH) terms used for extraction were *heart failure* OR *heart failure, congestive* OR *heart failure with reduced ejection fraction*. Terms like *left sided heart failure* OR *systolic heart failure* were not included to keep the search strategy easily replicable and clear. Studies were reviewed individually and screened by title and abstract. Sub-studies or post-hoc analyses were excluded. Trials looking at heart failure with preserved ejection fraction were excluded.

Data are presented using descriptive statistics frequencies and percentages. Mean and standard deviation were used while median interquartile range was used for skewed variables. The percentage of females included in studies with studies per year was log transformed and fitted into a time regression model by year. The Mann Whitney U test was used to compare the median percentages of females according to study size (more than 100 samples included and less than 100 samples). A p-value of 0.05 was considered statistically significant.

## Findings

**Figure 1:**
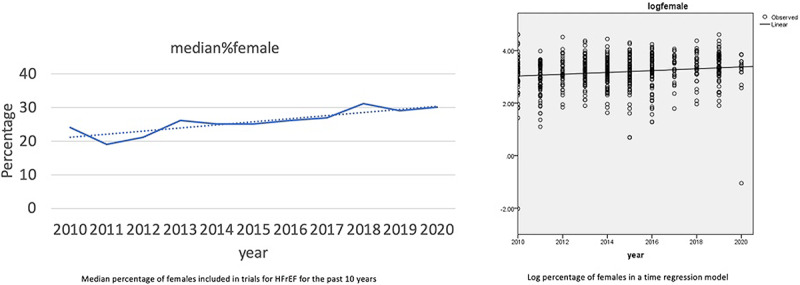
Shows a graph (left) depicting median percentage of female included in HFrEF trials in the past 10 year and log percentage of females in a time regression model.

A total of 3052 studies were reviewed, and 706 studies met criteria for inclusion. Thirty-seven percent of studies involved pharmacologic interventions, while 10% of studies looked at device therapy (implantable cardioverter and defibrillator/cardiac resynchronization therapy). The rest of the studies included a combination of exercise, community programs, surgical, procedural and laboratory testing. The pooled study sample age ranged from 42 to 85 years. Only 26% (17–36) of the participants were women. There were no significant differences in representation of women (%) across the nature of interventions (pharmacotherapy, device, other interventions). The median percentage of females included in trials of HFrEF over the past 10 years was less than 50% per year, but with a gradual increasing trend towards the later part of the decade p < 0.001 for linear trend, as shown in ***Figure 1***.

On the other hand, the median percentage of women recruited in RCTs for HFrEF was significantly higher in studies with more than 100 subjects 28 (22–37) versus 24 (14–36); (p < 0.001).

## Discussion

In this systematic review of 706 trials, we identified significant under-representation of women in HFrEF trials. This appears to be in line with the findings of similar analyses of heart failure trials [3], and acute coronary syndrome (ACS) trials (26.8% of women subjects) [4]. Under-enrolment of women puts the generalizability of these trials’ findings into question, as it is well known that there are sex-based differences in heart failure aetiologies, co-morbidities, prognosis as well as responses to treatments [5]. It is, however, reassuring to some extent that the recruitment of female participants has improved towards the later part of the decade. This reflects the efforts made towards ensuring gender equity in trial enrollment in the last decades by the federal government, Food and Drug Administration, professional societies, and the pharmacological and device industries. More efforts are needed in raising awareness and identifying potential barriers to recruitment of women in cardiovascular disease, to ensure adequate powering of RCTs to detect sex-based interactions and enable appropriate application of trial data to real-world practice. We acknowledge that our study did not include extraction of data from Databases like Cochrane or Embase, or non-PubMed indexed trials; however, we believe we still have provided an exhaustive review of a lot of studies across 10 years which would be able to present a picture of current disparity in heart failure trials.
